# Prognostic relevance of treatment deviations in children with relapsed acute lymphoblastic leukemia who were treated in the ALL-REZ BFM 2002 study

**DOI:** 10.1038/s41375-024-02474-6

**Published:** 2024-12-11

**Authors:** Eleni A. Argyriadi, Ingo G. Steffen, Christiane Chen-Santel, Andrej Lissat, Andishe Attarbaschi, Jean-Pierre Bourquin, Guenter Henze, Arend von Stackelberg

**Affiliations:** 1 Department of Pediatric Oncology Hematology, Charité- Universitätsmedizin Berlin, Berlin, Germany; 2 Universitätsklinikum Leipzig, Klinik und Poliklinik für Kinder- und Jugendmedizin, Abteilung für Pädiatrische Onkologie, Hämatologie und Hämostaseologie, Leipzig, Germany; 3 Department of Pediatric Hematology and Oncology, St. Anna Children’s Hospital, Medical University of Vienna, Vienna, Austria; 4 Department of Pediatric Oncology, University Children’s Hospital, Zurich, Switzerland

**Keywords:** Risk factors, Phase III trials

## Abstract

Relapsed Acute Lymphoblastic Leukemia (ALL) is among the most common causes of cancer-associated deaths in children. However, little is known about the implications of deviations from ALL treatment protocols on survival rates. The present study elucidates the various characteristics of treatment deviations in children with relapsed ALL included in the ALL-REZ BFM 2002 (i.e., Relapse Berlin-Frankfurt- Münster) trial and determines their prognostic relevance for relapse and death rates. Among 687 patients, 100 were identified with treatment deviations, further classified, and examined by occurrence time, cause and type. Protocol deviation was considered a time-dependent variable and its impact on Disease Free Survival (DFS) and Overall Survival (OS) was examined using the time-dependent model Mantel Byar. Five years after the relapse diagnosis, deviations were significantly related to both inferior DFS (38%) and OS (57%) rates compared to protocol conformed treatment (DFS = 61%; OS = 70%, *P* < 0.001). Based on multivariate analyses, protocol deviation proved to be an independent adverse prognostic factor of DFS. Moreover, deviations triggered by chemotherapy-induced toxicity were associated with a higher relapse rate compared to deviations due to insufficient response. Therefore, to avoid impairment of results by deviations, future clinical trials, and treatment strategies should focus on less toxic treatments and stricter protocol compliance.

## Introduction

Over the last 40 years, significant progress has been achieved in the treatment of childhood acute lymphoblastic leukemia (ALL), with long-term survival rates reaching 90% [1–4]. However, about 15% of children with ALL still relapse. The success of the treatment in children with relapsed ALL remains unsatisfactory. Despite modern protocols, post-relapse survival outcome ranges from 35 to 70% depending on the risk profile of the investigated patient cohort [5–8].

Since 1983 the Berlin-Frankfurt-Münster Group’s Acute Lymphoblastic Leukemia-Relapse Study (ALL-REZ BFM) has conducted consecutive national and international trials in order to develop and optimize treatment protocols for children with ALL relapse [2, 5].

In this study, we refer to the ALL-REZ BFM 2002 trial, in which patients were divided into 4 risk-adapted strategic treatment groups (S1 to S4) and received a risk-adapted combination of chemotherapy, allogeneic hematopoietic stem-cell transplantation (HSCT) and radiotherapy [9–11]. Over the course of this trial, two-thirds of the children survived and more than half of them remained in 2nd complete remission (CR) without experiencing a subsequent event [12]. Treatment failure was most likely in high-risk (HR) subgroups (S3/S4), with relapse being the most frequent event [2, 13].

Although treatment protocols for relapsed ALL are intended to be strictly followed, protocol deviations while on treatment within clinical trials were regularly documented. Although deviations could be a relevant parameter for the treatment outcome, only a few reports on the incidence and prognostic significance are available [14–17]. Goal of this retrospective study was to identify the characteristics of treatment deviations from the ALL-REZ BFM 2002 protocol and to estimate their prognostic relevance for patients suffering from relapsed leukemia.

## Methods

### Patients

Between August 2003 and June 2011, children up to the age of 18 years with 1st relapse of ALL were registered from participating centers in Germany, Austria, and Switzerland in the international, cooperative, randomized multicenter trial ALL-REZ BFM 2002 after approval by each local ethics committee (e.g., Charite, Humboldt University, Berlin - Approval number 222/2001). Written informed consent was obtained from guardians and patients according to the Declaration of Helsinki [11].

This retrospective study reports on all 687 patients that were registered in the ALL-REZ BFM 2002 trial and fulfilled the following eligibility criteria: age up to 18 years and a morphologically confirmed diagnosis of first relapse of ALL.

### Definitions

Six months before the completion of the primary therapy, relapse was classified as “very early” or “early” when diagnosed in less or more than 18 months after the initial ALL diagnosis respectively, whereas “late” relapse occurred 6 months after the completion of primary treatment. Isolated bone marrow (BM) relapse was defined as no extramedullary involvement and ≥25% blasts in the BM. Isolated extramedullary and combined BM relapse were described as involvement of extramedullary sites with <5% and ≥5% blasts in the BM, respectively. If cerebrospinal fluid (CSF) cytology revealed a pleocytosis of ≥5/µl in the CSF and leukemic blasts (assessed by light microscopy), a CNS relapse was diagnosed. A unilateral or bilateral painless testicular enlargement with leukemic blasts in the biopsy indicated a testicular relapse [11]. Both time point and site of relapse, as well as ALL immunophenotype and minimal residual disease (MRD) load at the end of the induction, were established risk factors predicting the prognosis of the disease. Based on the first three above, strategy groups were defined as standard (S1), intermediate (S2), and HR (S3/S4), respectively (Supplementary Table 1) [6, 7, 18–20]. Diagnostic tests like cytomorphology, immunophenotyping, and molecular genetic screening were conducted as described elsewhere [21, 22].

### Protocol design

Within the ALL-REZ BFM 2002 trial, cytoreductive pre-phase including dexamethasone was followed by the induction blocks F1 and F2. During the early consolidation phase, patients were assigned to receive either protocol II-IDA (arm A) or 3 alternating R blocks (arm B) randomly. Patients not participating in the randomization received arm A and were included in the study to investigate whether the randomization status had an impact on the present analysis.

Subsequently, late consolidation consisted of three R blocks for S1 patients and HSCT for HR patient group S3/S4, respectively. Patients of S2 risk group received further chemotherapy (R blocks) in case of MRD good response or HSCT in case of MRD poor response as consolidating strategy. Following prophylactic CNS radiotherapy in patient groups S1 and S2, oral continuation therapy was carried out over 1 year (S1) or 2 years (S2) [23]. The complete treatment design for groups S1–S4 is shown in Supplementary Fig. 1 [11].

All patients received intrathecal combination chemotherapy (MTX, cytarabine, and prednisolone) applied at each treatment element. In case of a CNS relapse, children received craniospinal radiotherapy and additional doses of intrathecal injection on day 6 of block F1, as well as on day 5 of each block R2 and day 8 of protocol II-IDA. The recommended dose depended on both age and previous radiation exposure [23, 24].

Regarding testicular relapse, orchiectomy was recommended for the clinically involved testis. A contralateral not involved testis was irradiated with 18 Gy if biopsy was positive and with 15 Gy if negative. Clinically affected, not removed testes were irradiated with a dose of 24 Gy.

### Therapy response

CR was defined as <5% blasts in the regenerating bone marrow and no further evidence of extramedullary disease. If CR was not achieved by the beginning of the fifth block (second block R2) or by the 29th day of protocol II-IDA (timepoint of response assessment), patients were considered non-responders. Relapse was defined as the reappearance of ≥25% lymphoblasts in BM or extramedullary manifestations at any site after achieving CR.

### MRD detection

In addition to the cytomorphological evaluation, detecting the leukemia load in a submicroscopic range (MRD) was crucial for the prognosis of the disease. The quantification of MRD was based on immunoglobulin and T-cell receptor gene rearrangements. An MRD load ≥10^−3^ after induction was defined as positive (or poor MRD response). Patients with late BM relapse of BCP ALL and MRD poor response were stratified to undergo allogeneic HSCT, whereas those with MRD <10^−3^ received further consolidation and continuation chemotherapy based on their chemotherapy-sensitive leukemia relapse [19, 20, 25–27]. During the study, MRD was not only measured after the second block but also during consolidation and before transplantation. However, the results were not supposed to be disclosed to the treating investigators and it was not planned to react based on these results. Therefore, disclosure of the MRD results upon request and any reaction to this was considered as protocol deviation.

### Protocol deviations

All clinical information related to this study was based on patient records, where documented deviations from the protocol were identified. Subsequently, protocol deviations have been categorized according to their cause, type, and occurrence time (Supplementary Table 6). The first cause of protocol deviation was patients with insufficient response due to persisting blasts after induction according to cytomorphological evaluation (i.e., ≥5% leukemic blasts in the ΒΜ after F2 block; not meeting the protocol definition of nonresponse), or positive MRD (remaining blasts after submicroscopic evaluation) during consolidation. Deviations due to toxicity of the protocol formed the second group of deviation cause, whereas the third group included other reasons such as family and PI decisions without a clear medical need, logistic causes, and unknown reasons.

Five different types of deviations have been identified in total: modification of the cytoreductive pre-phase, modification of the order of treatment courses, preterm termination of the intensive chemotherapy (Supplementary Table 5), therapeutic interventions based on unfavorable MRD results, as well as modification of the radiation dose.

Considering that deviations occurred during the course of the trial (induction, consolidation, or continuation phase), they were analyzed as a time-dependent variable in the statistical methods. Similarly, deviations prior to response evaluation were analyzed concerning their impact on the remission rates.

### Statistical analysis

All protocol deviations were investigated for their influence on disease-free survival (DFS) and overall survival (OS). DFS, which was the primary outcome of this study, was defined as the time between remission and the date of the last follow-up or the first event (i.e., second relapse, therapy-related death, and second malignancy), whereas secondary outcomes were OS and remission rate. The probability of OS (pOS) was calculated from the date of first relapse diagnosis until any death occurrence. Lost to follow-up patients were censored at the time of the last observation. The Mantel Byar test and the Simon Makuch plot were both used to estimate the DFS or OS probabilities, including deviation as a time-dependent variable; this method accounted for patients moving from one group to another [28–32]. The time-dependent dataset was developed using the *tmerge* function, as described elsewhere [32]. Furthermore, for the comparison of subgroups, the two-sided log-rank test was utilized. A two-sided *P* value less than 0.05 was considered significant throughout the study. In order to test whether variables were affecting independently the outcome, a multivariate Cox stepwise forward conditional regression analysis and a forward Wald test were used [33]. Assessment of the association of deviations prior to response evaluation with the remission rates was based on uni- and multivariate logistic regression models, including protocol deviation as a time-dependent covariate. HRs were given with 95% confidence interval (CI). Both *χ* ^*2*^ and Fisher’s exact tests were used to investigate differences in the distribution of categorical characteristics among different groups. Differences in cumulative incidence of deviations (CID) between subgroups were tested according to Gray’s test [27, 28]. All the above analyses were conducted by utilizing the R statistical computing software (version 4.2.3; http://www.r-project.org) [34].

## Results

### Patient characteristics

Of the 687 patients eligible for the present study, 437 (64%) participated in the randomization process, receiving either protocol II-IDA or 3 alternating R blocks (Supplementary Fig. 2). A total of 603 patients (88%) reached CR, whereas 84 patients (12%) were non-responders or died during induction. Out of all patients who reached CR, 94 (16%) of them experienced deviations from the treatment protocol, while 6 patients (7%) out of these who did not achieve CR underwent deviations during induction. Overall, 587 (85%) patients received the protocol treatment, whereas 100 (15%) experienced protocol deviations (Supplementary Fig. 2). Baseline characteristics of these two groups are depicted in Table 1. The distribution of gender, age, timepoint of relapse, randomization status and immunophenotype did not reveal a statistically significant difference between both groups (*p* > 0.05). Patients with isolated BM relapse were overrepresented in the deviation group (72%) compared to the protocol group (60%), without reaching statistical significance (*p* = 0.09). A higher proportion of children who experienced deviations had Down syndrome (DS) compared to the ones receiving protocol therapy (10% vs. 2%, *p* < 0.001). Furthermore, the rate of children experiencing deviations was higher in HR patients than in standard-risk patients (20% vs. 12%, *p* = 0.006).

**Table 1:** Demographics, baseline patient characteristics and outcome.

Patient characteristics						Outcome		
			Without protocol deviation	With protocol deviation		pDFS		
		No. of patients^a^ (%)	No. (%)	No. (%)	*P*(x^2^)	5y.	SE	*P*(log rank)
**All patients**		687(100)	587 (100)	100 (100)				
**Sex**	Male	419 (61)	362 (62)	57 (57)	0.508	0.5	0.45–0.55	0.5
	Female	268 (39)	225 (38)	43 (43)		0.52	0.46–0.59	
**Age at relapse (y)**	<10	414 (60)	360 (61)	54 (54)	0.244	0.51	0.46–0.56	0.9
	≥10 to <18	273 (40)	227 (39)	46 (46)		0.51	0.50–0.58	
**Randomized**	No	250 (36)	209 (36)	41 (41)	0.318	0.45	0.39–0.52	0.02
	Yes	437 (64)	378 (64)	59 (59)		0.54	0.50–0.60	
**Relapse time point**	Late	339 (49)	299 (51)	40 (40)	0.104	0.66	0.61–0.72	<0.001
	Early	199 (29)	168 (29)	31 (31)		0.43	0.36–0.51	
	Very early	149 (22)	120 (20)	29 (29)		0.2	0.19–0.34	
**Immunophenotype**	T cell	83 (12)	68 (11)	15 (15)	0.601	0.24	0.16–0.36	<0.001
	Non-T cell	531 (77)	456 (78)	75 (75)		0.56	0.51–0.60	
	No data	73 (11)	63 (11)	10 (10)		0.45	0.33–0.62	
**Site of relapse**	Isolated BM	426 (62)	354 (60)	72 (72)	0.09	0.51	0.46–0.56	0.5
	Combined BM	127 (18)	113 (19)	14 (14)		0.53	0.45–0.63	
	Isolated EM	134 (20)	120 (21)	14 (14)		0.47	0.39–0.58	
**Down syndrome**	No	663 (97)	573 (98)	90 (90)	<0.001	0.53	0.50–0.57	0.3
	Yes	24 (3)	14 (2)	10 (10)		0.48	0.30–0.75	
**Strategic group**	S 1/2	463 (67)	408 (70)	55 (55)	0.006	0.61	0.56–0.66	<0.001
	S 3/4	224 (33)	179 (30)	45 (45)		0.3	0.25–0.37	
**Outcome**	Early death without CR	19 (3)	16 (3)	3 (3)^b^	<0.001			
	Nonresponder	65 (9)	62 (11)	3 (3)^b^				
	TRD/unknown cause of death	37 (5)	31 (5)	6 (6)				
	Second mal.	22 (3)	17 (3)	5 (5)				
	Second relapse	183 (27)	141 (24)	42 (42)				
	CCR/LFU	361 (53)	320 (54)	41 (41)				

*pDFS* probability of disease-free survival at 5 years, *SE* standard error, *BM* bone marrow, *EM* extramedullary, *CCR* continuous complete Remission, *LFU* lost to follow up, *TRD* treatment related death, *CR* complete Remission.

^a^Non responders are included.

^b^Related to the total group of deviations and needs to be interpreted in context with the time-dependency.

### Deviation prior to response evaluation

Supplementary Table 3 illustrates the investigation of the influence of deviations prior to response evaluation on the remission rates. Among 30 patients with deviation before response assessment, 24 patients achieved remission (24/30, 80%), whereas among 657 patients without deviation prior to response evaluation, 579 achieved remission (579/657, 88%; *P* = 0.39). Therefore, there was no significant association between the protocol deviation prior to response evaluation and the remission status.

### Protocol deviation: influence on disease-free and overall survival

**Fig. 1:**
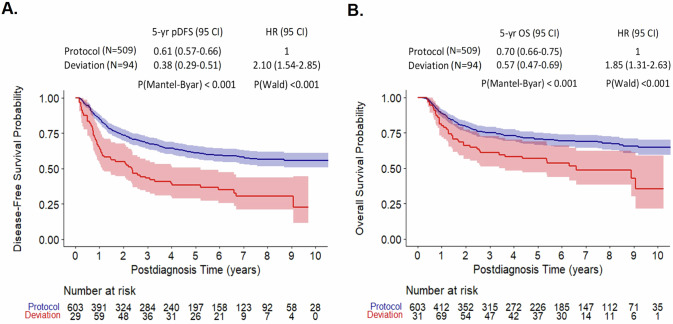
Clinical outcomes following deviations of the ALL-REZ BFM 2002 protocol. Mantel Byar analyses; Simon Makuch plots of Disease-free survival (**A**) and Overall Survival (**B**) of patients with versus without protocol deviations treated in the ALL-REZ BFM 2002 trial. In the Mantel Byar statistics, the initial number of patients at risk may be higher than the total patient sample, mainly due to patients being counted twice while shifted from the protocol to the deviation group during the respective observation period. pDFS probability of disease-free survival, OS overall survival, 95CI 95% Confidence Interval, HR hazard ratio.

In the present time-dependent analysis, the probability of DFS (pDFS) five years after the relapse diagnosis was significantly lower in patients with protocol deviations (*n* = 94; pDFS = 0.38 (0.29–0.51)) than those without (*n* = 509; pDFS = 0.61 (0.57–0.66); *P*(Mantel Byar) < 0.001, Fig. 1A). Similarly, the pOS in the deviation group was significantly inferior to those of protocol group (*P*(Mantel Byar) < 0.001, Fig. 1B). Patients with deviations were at higher risk of relapse (HR = 2.10, 95% CI: 1.54–2.85, *P*(Wald) < 0.001) but also at higher risk of death (HR = 1.85, 95% CI: 1.31–2.63, *P*(Wald) < 0.001).

### Cumulative incidence of deviations

**Fig. 2:**
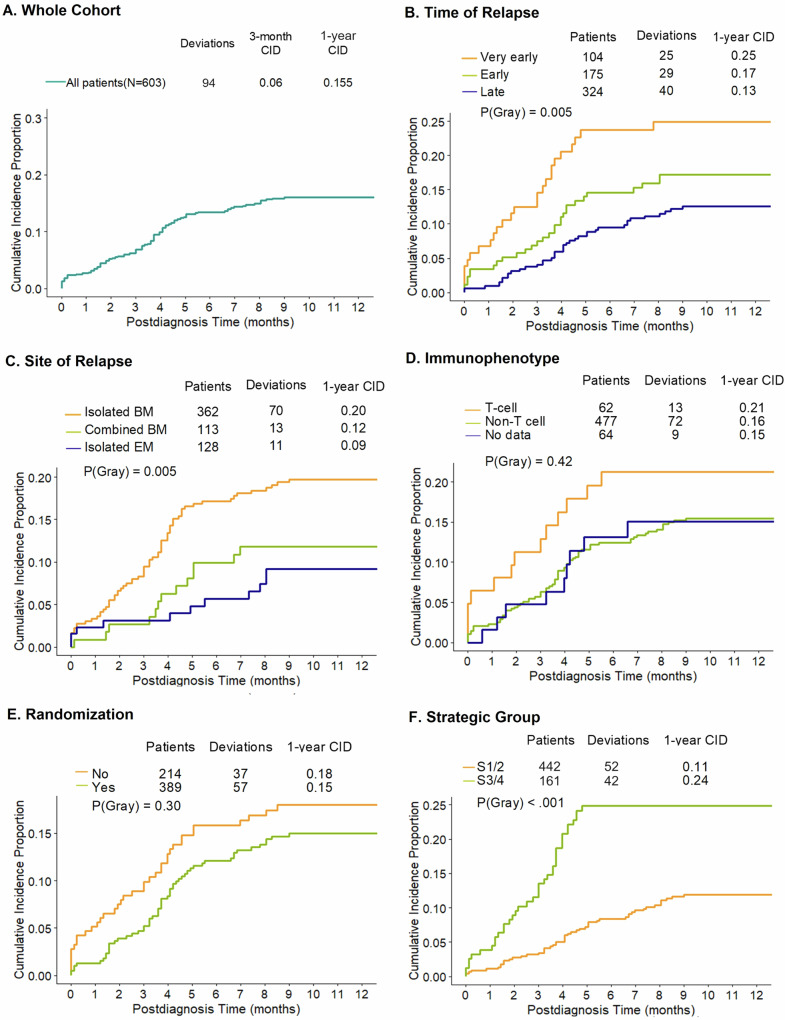
Cumulative incidence of deviation (CID) in patients of the BFM group. Data are shown (**A**) in the whole cohort, (**B**) in time of relapse groups, (**C**) in site of relapse groups, (**D**) in immunophenotype group, (**E**) in randomization groups, and (**F**) in strategy groups (S1/2, S3/4). BM bone marrow, EM extramedullary.

Moreover, we analyzed the CID of deviations in the total group as well as the clinically relevant subgroups. The 3-month and 1-year cumulative incidences of protocol deviation in the whole cohort were 0.06 and 0.155, respectively (Fig. 2A). Based on the three time of relapse groups, the CID among patients with very early relapse was 0.25 at first year and thus significantly higher than in the cohort with early (CID = 0.17) and late relapse (CID = 0.13; *p*(Gray) = 0.005, Fig. 2B). Between the three site of relapse groups, the CIDs at first year were significantly different (CID BM: 0.20, CID EM: 0.09 and CID Combined BM: 0.12, *p*(Gray) = 0.005, Fig. 2C). Gray’s test showed also that there was no significant difference in CID between B- and T-cell immunophenotype as well as between the randomization groups (*p*(Gray) = 0.42, Fig. 2D; *p*(Gray) = 0.30, Fig. 2E). Overall, significantly fewer standard risk patients (strategic groups S1/2) underwent protocol deviations compared to HR patients (strategic groups S3/4; *p*(Gray) < 0.001, Fig. 2F).

### Multivariate analysis

A univariate analysis confirmed that time point of relapse, ALL immunophenotype, randomization status, and protocol deviation status had all significant impact on pDFS (*p* < 0.01, Table 2). In a time-dependent multivariate Cox regression analysis, deviation status, time of relapse, and immunologic subtype were all found to be independent predictors of pDFS (*p*(Wald) ≤ 0.001, Table 2). In the analysis above, patients with protocol deviations had a hazard ratio of suffering a subsequent adverse event of 1.71 (95% CI: 1.24–2.36, *p* = 0.001) compared to patients without deviations. The rest of the factors included (i.e., sex, DS, age at relapse, randomization status, and site of relapse) did not significantly improve the model.

**Table 2:** Uni- and multivariate Cox proportional regression analysis for DFS.

Variable	No. (%)	Univariate analysis			Multivariate analysis		
		HR	95% CI	*P*-Value	HR	95% CI	*P*-Value
**Protocol deviation**^a^				<0.001			0.001
No	509 (84)	1			1		
Yes	94 (16)	2.1	1.55–2.85		1.71	1.24–2.36	
**Sex**				0.6			0.69
Male	366 (61)	1			1		
Female	237 (39)	0.93	0.72–1.20		1.05	0.80–1.38	
**Down syndrome**				0.4			0.59
No	583 (97)	1			1		
Yes	20 (3)	1.32	0.67–2.57		1.23	0.58–2.58	
**Age at relapse(y)**				0.063			0.96
≤5	106 (18)	1			1		
>5 to ≤10	262 (43)	0.67	0.47–0.94		0.99	0.62–1.57	
>10 to ≤14	127 (21)	0.62	0.41–0.92		0.97	0.58–1.60	
>14 to ≤18	108 (18)	0.8	0.53–1.20		1.07	0.64–1.80	
**Randomization**				0 .007			0.19
No	214 (35)	1			1		
Yes	389 (65)	0.7	0.54–0.91		0.83	0.62-–1.10	
**Relapse time point**				<0.001			<0.001
Late	324 (54)	1					
Early	175 (29)	2.12	1.59–2.83		1.97	1.43–2.70	
Very early	104 (17)	3.5	2.53–4.86		2.73	1.87–4.00	
**Immunophenotype**				<0.001			0.001
T cell	62 (10)	1			1		
Non-T cell	477 (79)	0.38	0.27–0.53		0.5	0.35–0.73	
No data	64 (11)	0.53	0.32–0.88		0.69	0.40–1.17	
**Site of relapse**				0.185			0.95
Isolated BM	362 (60)	1			1		
Combined BM	113 (19)	0.9	0.64–1.26		0.95	0.66–1.36	
Isolated EM	128 (21)	1.26	0.93–1.73		0.96	0.67–1.36	

*DFS* disease-free survival, *95% CI* 95% confidence interval, *BM* bone marrow, *EM* extramedullary.

^a^Protocol Deviation considered as time-dependent variable.

### Impact of deviation per time-point of relapse groups

**Fig. 3:**
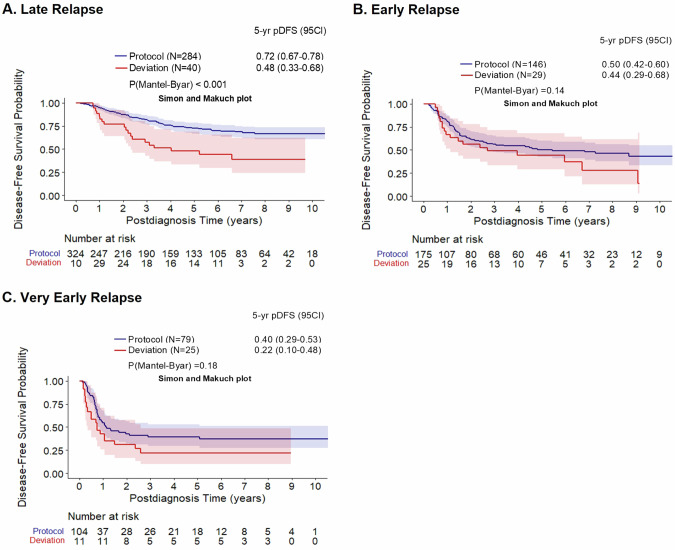
Clinical outcomes following deviations of the ALL-REZ BFM 2002 protocol in relation to time of relapse. Mantel Byar analyses; Simon Makuch plots of Disease-free survival and Overall Survival of patients with versus without protocol deviations based on the time of relapse groups of the the ALL-REZ BFM 2002 protocol; (**A**) Late; (**B**) Early; (**C**) Very Early. pDFS probability of disease-free survival, 95CI 95% Confidence Interval.

Analyzing the implications of protocol deviation on the prognosis in terms of time-point of relapse, revealed that the difference of pDFS between protocol and deviation patients was significant only for patients with late relapse (late relapse: pDFS deviation: 0.48 (0.33–0.68) vs. pDFS protocol: 0.72 (0.67–0.78), *P*(Mantel Byar) < 0.001, Fig. 3A). Instead, for patients with early and very early relapse, no significant prognostic effect could be identified with the current approach (Fig. 3B, C).

### Classification and description of deviations

Further analysis was performed according to cause, time of occurrence and type of deviation. Stratifying by cause, 40% of the patients underwent protocol deviation due to insufficient response (*n* = 37/94), 34% due to toxicity (*n* = 32/94) and 26% due to other reasons (*n* = 25/94) (Supplementary Table 6). At five years, the DFS rates were not significantly different between patients treated according to protocol guidelines and those experiencing protocol deviations caused by insufficient response (pDFS protocol: 0.61 (0.56–0.66) vs. pDFS deviation due to insufficient response: 0.49 (0.34–0.70), *p* = 0.09, Supplementary Fig. 3A). In contrast, those patients with treatment deviations due to toxicity and other reasons had significantly inferior pDFS compared to patients treated according to the trial protocol (pDFS protocol: 0.61 (0.56–0.66) vs. pDFS deviation due to toxicity: 0.36 (0.22–0.59), *p* < 0.001, pDFS deviation due to other reasons: 0.25 (0.11–0.54), *p* < 0.001). Similarly, the OS probabilities were comparable between patients treated on protocol and patients with deviations based on poor prognosis insufficient response group (Supplementary Fig. 3B).

Regarding the protocol phase, deviations were categorized into the following three groups: induction (*n* = 25), consolidation (*n* = 55) and continuation group (*n* = 14). The estimated 5-year probabilities of DFS and OS between the protocol patients and patients with deviations during induction were significantly different (*p*(Mantel Byar) < 0.001, Supplementary Fig. 4). Similarly, pDFS and pOS were significantly higher in protocol patients than in patients with deviation during consolidation (*p*(Mantel Byar) < 0.001, Supplementary Fig. 4). Deviations during continuation therapy (*n* = 14) concerned mostly unexpected modifications in the radiation plan. In detail, 9 patients did not receive CNS or other local radiation, 4 patients were given radiation beyond the protocol doses, and 1 patient underwent modifications of the radiation treatment due to unknown reasons (Supplementary Table 4). Deviations during continuation therapy were not associated with significantly decreased DFS (*p*(Mantel Byar) =  0.07) or OS (*p*(Mantel Byar) = 0.07).

Depending on the type of deviation, five distinct subgroups were created: modification of the cytoreductive prephase in 7% of patients (*n* = 7/94), modification of the order of protocol elements in 37% (*n* = 35/94), preterm termination of the intensive chemotherapy and mainly alternative continuation with continuation therapy in 11% (*n* = 10/94, Supplementary Table 5), intensification of the treatment due to positive MRD mostly during late consolidation in 31% (*n* = 29/94) and modifications in their radiation plan in 14% (*n* = 13/94, Supplementary Table 6). In a time-dependent model, both patients with intervention due to MRD and patients with changes in the radiation plan had similar 5-year pDFS and pOS compared to patients without any deviation (*p*(Mantel Byar) = 0.6, *p*(Mantel Byar) = 0.4, respectively; Supplementary Fig. 5). On the contrary, significantly lower pDFS and pOS were observed in patients belonging to the common group created for the rest three deviation types, modification of the order of protocol elements, termination of protocol treatment and modifications of the cytoreductive prephase compared to patients without deviation (*p*(Mantel Byar) < 0.01). Furthermore, the influence of the deviation on HSCT realization rates was analyzed in patients with indication for HSCT (S3/S4 and S2 patients with positive MRD). Among 221 patients without deviation, eligible for HSCT, 185 patients received stem cell transplantation (185/221, 83%), whereas among 60 patients with deviation, eligible for HSCT, 48 received stem cell transplantation (48/60, 80%; *P* = 0.72). Therefore, there was no significant correlation between the protocol deviation and the HSCT status in patients with HSCT indication.

## Discussion

The present analysis provides novel information on the identification, classification, and prognostic relevance of protocol deviations in pediatric patients with relapsed ALL; a special group of patients with an outstanding need for more effective and less toxic therapeutic options [5, 6]. Processing of the data revealed that deviations before response assessment had no impact on the remission rate. This study showed that patients who experienced treatment deviations achieved lower disease free- and overall-survival rates than patients who received therapy according to the protocol in the ALL-REZ BFM 2002 trial. Protocol deviation proved to be a significant prognostic factor for EFS and OS in multivariate analysis, independent from other known risk factors such as time of relapse and immunophenotype.

The prognostic relevance of protocol deviation was detected only in patients with late relapse. We hypothesize that early and very early relapse disease incorporate a comparable high level of chemotherapy resistance to a multitude of respective compounds, not leaving relevant room for the prognostic effect of treatment deviation.

The prognostic impact on outcome was highly significant in patients with deviations due to severe toxicity or other reasons, mostly leading to preterm termination of the intensive chemotherapy, dose reduction or treatment delay. Currently, these patients might be offered efficient immunotherapeutic alternatives including inotuzumab, blinatumomab and CAR-T, approaches that were lacking 15 years ago [35–38].

Insufficient response to induction or MRD persistence during early consolidation are well known adverse prognostic factors and both contribute to a negative selection of patients with treatment deviation, hence may reveal a bias when assessing the prognostic impact of the deviation itself. Therefore, we analyzed the causes for deviation separately. Nevertheless, deviation due to unfavorable MRD results led to off-protocol intensification of the treatment which seemingly at least in part could have rescued the adverse prognosis. However, there was no significant difference in DFS between patients with positive MRD post induction with and without MRD triggered intervention, therefore we cannot conclude from our data that the intervention led to a significant improvement of those patients’ outcome.

Insufficient response after induction or during consolidation frequently triggers individualized and mostly intensified treatment. To prevent this, international experts have recommended including cytological nonresponse at a threshold of 5% leukemic blasts in the bone marrow aspirate, MRD persistence and MRD relapse as primary endpoints in future trials. This approach aims to avoid protocol deviations and allows treatment intensification, ideally in a safe and controlled environment within open phase I/II trials [39]. Moreover, the design of protocol-based clinical decision support systems (data analysis applications that assist healthcare providers with decision making) might contribute to enhance compliance with chemotherapy protocols and patient safety [16].

The obtained results are in line with previous studies showing that interrupting the execution of protocols due to unpredictable conditions is accompanied by a significant worsening of long-term results of the treatment [16, 17, 40, 41]. On UKALL2003 trial, deviations during induction, especially dose reduction >90% of one or more drugs and a delay greater than 1 week in commencing the next treatment block, were strongly related to a higher probability of future deviations in subsequent blocks of therapy and a higher risk of relapse [14].

In our analysis, we found that the clinical and demographic characteristics of our study population were balanced between the deviation and non-deviation group, possibly indicating that the deviation group may not necessarily represent a less favorable subgroup with more aggressive disease. Nevertheless, patients with DS and HR patients were overrepresented in the deviation group. We assumed that the DS patients undergo more protocol deviations because of their decreased tolerance to the treatment protocol due to a relative lack of prognostically favorable cytogenetic risk groups in DS-ALL [42–44]. However, in the multivariate analysis, the covariate DS did not reach independent significance and did not impair the prognostic relevance of treatment deviation.

The remission rate was expected to be lower in the deviation group than in the protocol group. Our study, on the contrary, revealed that the remission rate was not significantly inferior among the patients with deviation compared to the protocol group. A possible explanation is that the time from deviation until response evaluation was too short and that the adverse prognostic impact took more time until consolidation to develop.

Among patients with deviations of the radiation plan, no statistically significant DFS was observed among the protocol and non-protocol group during continuation. This finding might be due to the small number of observations, the heterogeneity of the deviations including either more or less irradiation than suggested by the protocol and the rather late time-point of deviation.

Considering the influence of cause and type of deviation, toxicity seems to be a major factor causing deviation which respectively results in impaired survival. Latest studies focus on the discovery of less toxic and ideally targeted treatment strategies, aiming for a better prognostic outcome with reduced toxicity, resulting in less treatment deviations. Furthermore, the third cause of deviation which was mainly related to decisions of the doctors or the families without clear medical reason might be reduced by clear statements on the protocol that deviation is related to a worse prognosis and on the other side with a better central and local monitoring to maintain strict protocol compliance as much as possible.

The present study demonstrates the importance of documentation and analysis of deviations from study protocols. Protocol deviation is a relevant parameter assessing the feasibility of a proposed treatment protocol. The endpoints of the trial should be defined in a way that insufficient response is considered an endpoint too, allowing for adequate intensification instead of keeping the patient in the trial and risking deviations from the protocol initiated by the treating PI’s.

The interpretation of this study is limited due to its retrospective character and furthermore due to the fact that in 11% of patients, immunophenotype was missing. Other limitations of our study were the heterogeneity of deviations with low numbers in specific subgroups. We cannot assess the effect of the intervention due to insufficient response compared to patients without insufficient response because of the different time points and the different MRD levels. Furthermore, HR genetics have not been documented in this study. Therefore, their potential impact on the results cannot be addressed in our analysis [45]. Moreover, several host factors related to toxicity, such as age, sex and DS, have been investigated with respect to protocol deviation. Other host factors such as TPMT deficiency have not been routinely investigated and cannot be therefore examined in the study.

In summary, the current analysis demonstrates that protocol deviations on the ALL-REZ BFM 2002 trial were significantly associated with a greater risk of relapse. Serious toxicity during the protocol treatment proved to be among the most significant causes of deviation related to inferior survival. In recent years, there have been efforts to minimize toxicity by including immunotherapeutic approaches in protocol therapies and more efficient supportive care. Future research should consider the significance of protocol compliance, while developing even less toxic treatment strategies.

## Supplementary information

Supplementary Information 1

Supplementary Information 2

## Data Availability

The datasets generated and analyzed during the current study are available from the ALL REZ Working Group via the corresponding author upon reasonable request.
