# Supplementary material for: Prognostic relevance of treatment deviations in children with relapsed acute lymphoblastic leukemia who were treated in the ALL-REZ BFM 2002 study
Source: Leukemia. 2024 Dec 11;39(2):337–45. doi: 10.1038/s41375-024-02474-6 (PMC11794146; doi:10.1038/s41375-024-02474-6)
Supplement: Supplementary file 1 — Supplementary Information 1 [file 41375_2024_2474_MOESM1_ESM.pdf]

Supplementary Information

Prognostic relevance of treatment deviations in children with relapsed acute lymphoblastic leukemia who were treated in the ALL-REZ BFM 2002 study

Authors: Eleni A. Argyriadi[1✉], Ingo G. Steffen[1], Christiane Chen-Santel[2], Andrej Lissat[1], Andishe Attarbaschi[3], Jean-Pierre Bourquin[4], Guenter Henze[1], Arend von Stackelberg[1]

[1]Department of Pediatric Oncology Hematology, Charité- Universitätsmedizin Berlin, Berlin, Germany. [2] Universitätsklinikum Leipzig, Klinik und Poliklinik für Kinder- und Jugendmedizin, Abteilung für Pädiatrische Onkologie, Hämatologie und Hämostaseologie, Leipzig, Germany. [3]Department of Pediatric Hematology and Oncology, St. Anna Children's Hospital, Medical University of Vienna, Vienna, Austria. [4]Department of Pediatric Oncology, University Children's Hospital, Zurich, Switzerland. ✉email: eleni.argyriadi@charite.de. Competing interests: EA, IS, GH, CC-S, AL, AA, JPB have no conflict of interest to declare. AvS declares consulting role and honoraria from Amgen, Novartis, Jazz, Miltenyi

This file contains information on the treatment protocol design and the enrollment of patients in the current study. Comparisons of the different categories of deviations are presented. Five-year OS and DFS of patients with versus without protocol deviations (in terms of cause, type and occurrence time of the deviation) are displayed along with 95% confidence intervals.

Supplementary Methods

Supplementary Definition: Protocol ALL-REZ BFM 2002 conform adjustments. *3*

Supplementary Tables

Supplementary Table 1: Strategy groups S1 to S4 of patients in ALL-REZ BFM 2002 trial. *3*

Supplementary Table 2: Classification and description of deviations of ALL-REZ BFM 2002 trial: Number of deviations by type and reason. *3*

Supplementary Table 3: Influence of deviations before response evaluation on the remission rates in patients of ALL-REZ BFM 2002 trial. *4*

Supplementary Table 4: Different ways of change of the radiation plan of the protocol ALL REZ BFM 2002. *4*

Supplementary Table 5. Preterm termination of the intensive chemotherapy. *5*

Supplementary Figures

Supplementary Figure 1: Treatment design of trial ALL-REZ BFM 2002. *6*

Supplementary Figure 2: Flow diagram of enrolment and analysis for study patients. *6*

Supplementary Figure 3: Mantel Byar analyses; Simon Makuch plots of Disease-free survival (A) and Overall Survival (B) of patients in the trial ALL-REZ BFM 2002 in relation to the cause of deviation. *7*

Supplementary Figure 4: Mantel Byar analyses; Simon Makuch plots of Disease-free survival and Overall Survival of patients with versus without protocol deviations in relation to the phase of the ALL-REZ BFM 2002 protocol. *8*

Supplementary Figure 5: Mantel Byar analyses; Simon Makuch plots of Disease-free survival (A) and Overall Survival (B) of patients in the trial ALL-REZ BFM 2002 in relation to the type of deviation. *9*

Supplementary Definition: Protocol ALL-REZ BFM 2002 conform adjustments

7.7 Reduction of the treatment intensity based on toxicity (page 61)

Modified WHO criteria for the classification of specific side effects are used to assess toxicity (table 13, p.62).

If the solid line demarcating the zone of dangerous toxicity is crossed during the preceding block or if the dashed line that demarcates the zone of alarming toxicity is crossed immediately prior to starting a new treatment element the following recommendations apply.

• in a subsequent **Block R1** cytarabine is reduced to 60% of the target dose and 6-mercaptopurine is administered at the original dose but only on day 1 to 3.

• in a subsequent **Block R2** ifosfamide and thioguanine are administered at the original dose but only on days 1 to 3.

The suggested approach attempts to accommodate the wide range of individual treatment toxicity. It is probable that not every situation that occurs in individual patients can be recorded and assessed in a standardized fashion. (1)

Supplementary Table 1: Strategy groups S1 to S4 of patients in ALL-REZ BFM 2002 trial

Immunophenotype	pB-ALL			T-ALL		
Time \ Site	Isolated EM	Combined BM	Isolated BM	Isolated EM	Combined BM	Isolated BM
Very early	S2	S4	S4	S2	S4	S4
Early	S2	S2	S3	S2	S4	S4
Late	S1	S2	S2	S1	S4	S4

Supplementary Table 2: Classification and description of deviations of ALL-REZ BFM 2002 trial: Number of deviations by type and reason

Reason vs. Type of deviation	Total (%)	Impaired Response	Toxicity	Other Reasons (parents,logistics,not found)
Modification of the Order of the Blocks	35 (37)	7 (19)	20 (63)	8 (32)
Preterm termination of the intensive chemotherapy	10 (11)	0 (0)	9 (28)	1 (4)
Prephase modification	7 (7)	1 (3)	1 (3)	5 (20)
Intervention due to positive MRD	29 (31)	29 (78)	0 (0)	0 (0)
Change of radiation	13 (14)	0 (0)	2 (6)	11 (44)
All patients	94 (100)	37 (100)	32 (100)	25 (100)

Supplementary Table 3: Influence of deviations before response evaluation on the remission rates in patients of ALL-REZ BFM 2002 trial

	Total (%)	Remission Number(%)	NR/ID Number(%)	Univariate OR	P-Value	Multivariate OR	P-Value
**Randomization**					0.16		0.42
No	250	214(86)	36(14)				
Yes	437	389(89)	48(11)	1.39		1.24	
**Relapse time point**					<0.001		<0.001
Late	339	324(96)	15(4)				
Early	199	175(88)	24(12)	0.33		0.24	
Very early	149	104(70)	45(30)	0.1		0.11	
**Immunophenotype**					0.008		0.07
T cell	83	62(75)	21(25)				
Non-T cell	531	477(90)	54(10)	3.04		2.33	
No data	73	64(88)	9(12)	2.4		2.01	
**Site of relapse**					0.002		<0.001
Isolated BM	426	362(85)	64(15)				
Combined BM	127	113(89)	14(11)	1.4		1.5	
Isolated EM	134	128(95)	6(5)	3.71		6.88	
**Deviation before response evaluation***					0.39		0.52
No	657	579(88)	78(12)				
Yes	30	24(80)	6(20)	0.64 (0.26-1.96)		1.47 (0.52- 4.94)	

NR=non response, ID=induction death, OR=odds ratio, *response evaluation= remission or non response or induction death, EM= extramedullary

Supplementary Table 4: Different ways of change of the radiation plan of the protocol ALL REZ BFM 2002

Change of the radiation plan	No CNS radiation	Other local radiation not received	Radiation beyond the protocol	Other	Total
Number of patients	8	1	4	1	14

Supplementary Table 5: Preterm termination of the intensive chemotherapy

Protocol phase	Reason of termination	Instead of intensive chemotherapy
Preterm termination of the intensive chemotherapy after 1st Block	Discontinuation of therapy due to massive toxicity = severe mucositis with corresponding pain symptoms, pancreatitis, hepatitis, sepsis, thrombocytopenia, anemia	Maintenance therapy with 6 mercaptopurine (MP) and methotrexate (MTX)
Preterm termination of the chemotherapy after 2nd Block	Septical shock with respiratory and cardiovascular arrest and cardiopulmonary resuscitation, encephalopathy due to hypoxia during resuscitation, with existing neurological symptoms, patient intubation and infection dose reduction of Prot II IDA Prots but not well tolerated, therefore termination of intensive chemo and beginning of maintenance therapy.	Maintenance therapy with 6MP and MTX
Preterm termination of the intensive chemotherapy after 3rd Block	Tuberkulosis	Anti TB treatment and maintenance therapy
Preterm termination of the intensive chemotherapy after 4th Block	Because of SAE : sepsis with gangrene of the ascending colon, emergency helikolectomy, ventilation in the intensive care unit	Maintenance therapy and HSCT with non-myeloablative conditioning
Preterm termination of the intensive chemotherapy after 5th Block	Because of SAE (colitis and bilateral preural effusions) termination of the protocol therapy	HSCT
Preterm termination of the intensive chemotherapy after 5th Block	Severe encephalopathy after a prolonged seizure with tetraspasticity and reduced vigilance. Due to the unclear neurological situation, the last two chemotherapies of the protocol were not administered and instead, the maintenance therapy was preferred and well tolerated for 2 years	Maintenance therapy with 6MP and MTX
Preterm termination of the intensive chemotherapy after 6th Block	SAE: sepsis after F1, fever in neuropenia after F2, pneumonia after R1, brain damage as a result of hypoxia during cardiopulmonary reanimation with cardiopulmonary decompensation during anesthetic initiation.	Maintenance therapy with 6MP and MTX
Preterm termination of the chemotherapy after 8th Block	Termination due to high therapy toxicity: neurological problems, seizures, double vision, consciousness, leukoencephalopathy, catheter sepsis, abscess in the area of the right thigh	No info
Preterm termination of the intensive chemotherapy during PROT II IDA	Intensive care unit because of respiratory insufficiency and sepsis.	LFU/ Parents decided to return to Moscow
Preterm termination of the intensive chemotherapy after Prot. II IDA	Down Syndrom	No info

Supplementary Figure 1

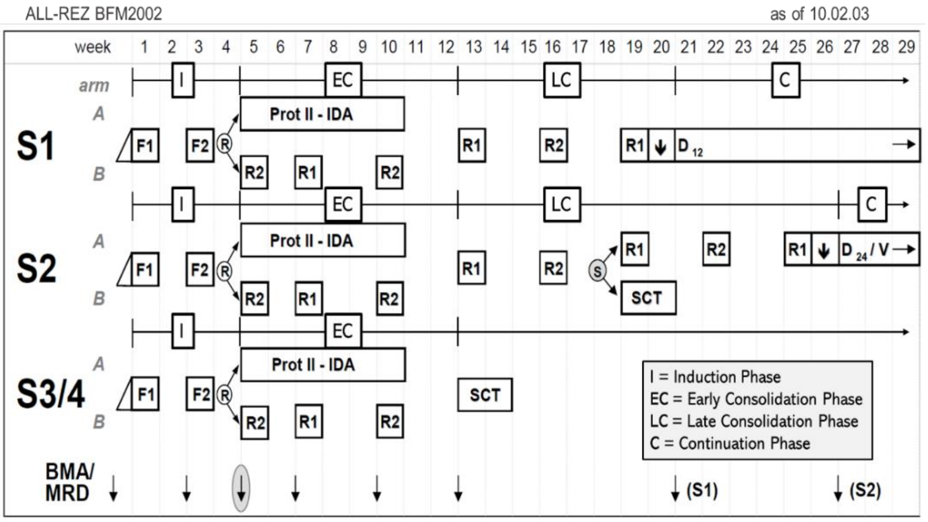

Supplementary Figure 1: Treatment design of trial ALL-REZ BFM 2002. D_{12}/D_{24}, 12/24 Months maintenance; Ⓡ, Randomization; Ⓢ, Stratification V, VP16 reinduction pulse; 🡻, Local radiation therapy; ⇣, BMP-Timepoint for postremission stratification in S2; SCT, Stem cell transplantation; BMP, Bone marrow puncture; MRD, minimal residual disease; Chemotherapy courses: F1, F2, R2, R1, Protocol ‖-IDA.

Supplementary Figure 2

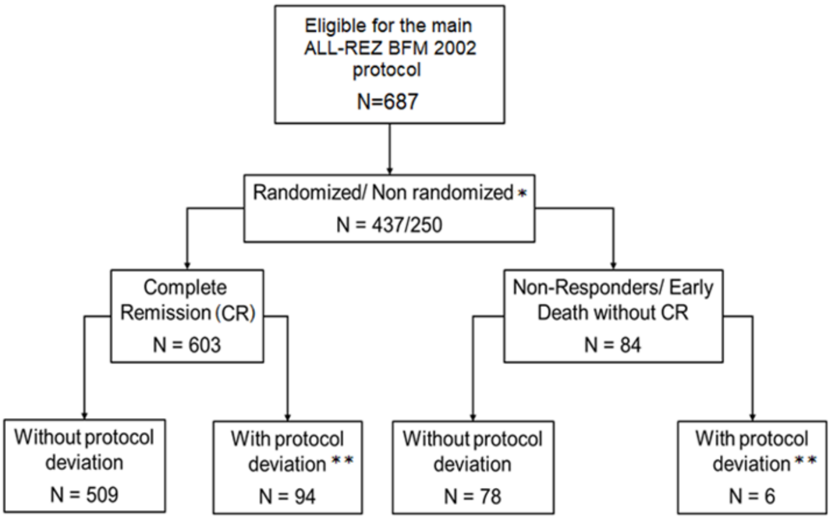

Supplementary Figure 2: Flow diagram of enrolment and analysis for study patients (up to 18 years with first relapse of ALL). CR; complete remission. *Not randomized for the study question R-courses versus protocol II-IDA. **The total number of patients with protocol deviation was 100 (1)

Supplementary Figure 3

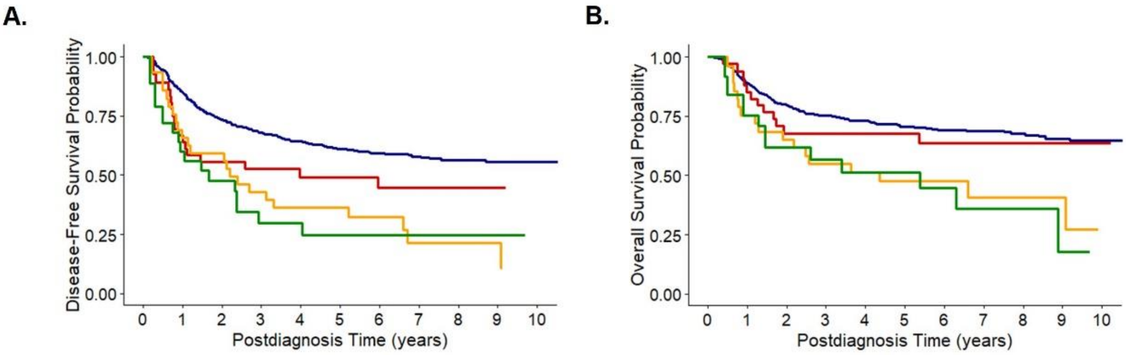

A. Number at risk

Protocol	603	391	324	284	240	197	158	123	92	58	28
Insuficient Response	14	24	19	17	14	13	10	4	3	1	0
Toxicity	15	20	18	13	11	9	8	3	3	2	0
Other	9	15	11	6	6	4	3	2	1	1	0

Reason	N	5-yr DFS (CI95)	HR (95CI)	P
No deviation	509	0.61 (0.56-0.66)	1.00	
Insuficient Response	37	0.49 (0.34-0.70)	1.54 (0.94-2.54)	0.09
Toxicity	32	0.36 (0.22-0.59)	2.45 (1.57-3.81)	<0.001
Other	25	0.25 (0.11-0.54)	2.53 (1.47-4.36)	<0.001

B. Number at risk

Protocol	603	412	352	315	272	226	185	147	112	71	35
Insuficient Response	33	30	22	21	19	18	14	5	4	2	1
Toxicity	17	22	19	16	14	11	10	5	4	3	0
Other	10	17	13	10	9	8	6	4	3	1	0

Reason	N	5-yr OS (CI95)	HR (95CI)	P
No deviation	509	0.70 (0.66-0.75)	1.00	
Insuficient Response	37	0.68 (0.54-0.85)	1.24 (0.68-2.23)	0.5
Toxicity	32	0.48 (0.33-0.70)	2.27 (1.37-3.75)	0.001
Other	25	0.51 (0.33-0.80)	2.38 (1.32-4.30)	0.004

Supplementary Figure 3: Mantel Byar analyses; Simon Makuch plots of Disease-free survival (A) and Overall Survival (B) of patients in the trial ALL-REZ BFM 2002 in relation to the cause of deviation; no deviation, insufficient response, toxicity and other reasons. pDFS, probability of disease-free survival; pOS, probability of overall survival; 95CI, 95% Confidence Interval; HR, hazard ratio

Supplementary Figure 4

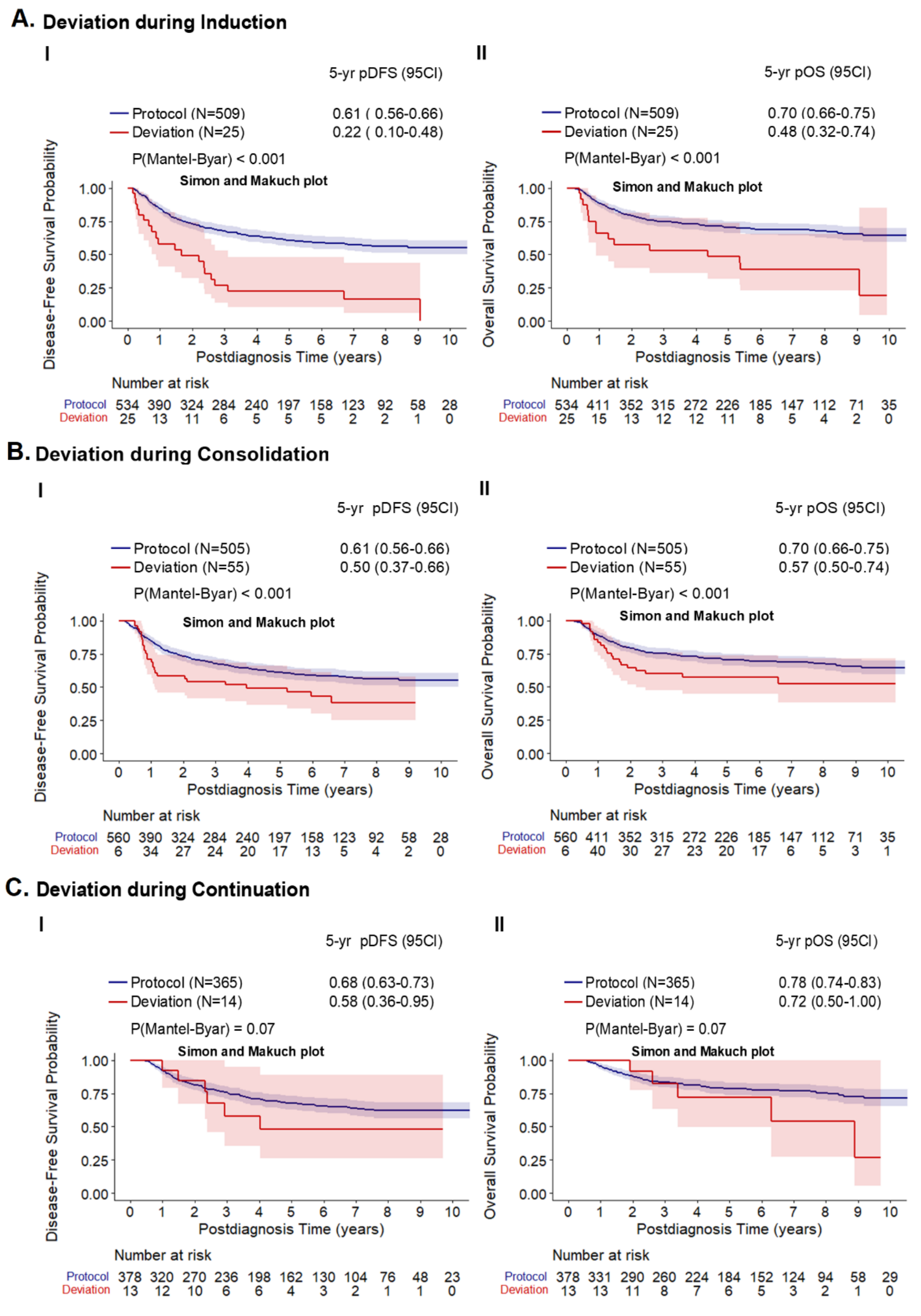

Supplementary Figure 4: Mantel Byar analyses; Simon Makuch plots of Disease-free survival and Overall Survival of patients with versus without protocol deviations in relation to the phase of the ALL-REZ BFM 2002 protocol; (A) Induction; (B) Consolidation; (C) Continuation. pDFS, probability of disease-free survival; pOS, probability of overall survival; 95CI, 95% Confidence Interval

Supplementary Figure 5

A.

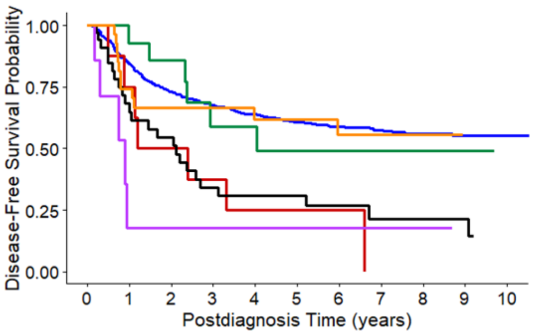

Number at risk

Protocol	603	391	324	284	240	197	158	123	92	58	28
Termination	4	6	4	3	2	1	1	0	0	0	0
Order Modification	20	20	16	10	9	8	7	3	3	3	0
Prephase Modification	7	1	1	1	1	1	1	1	1	0	0
Radiation	13	12	10	6	6	4	3	2	1	1	0
Intervention due to positive MRD	27	20	17	16	13	12	9	3	2	0	0

Type	N	5-yr DFS (CI95)	HR (95CI)	P
Protocol	509	0.61 (0.56-0.66)	1.00	
Termination	10	0.24 (0.07-0.82)	3.30 (1.54-7.02)	0.002
Order Modification	35	0.29 (0.17-0.51)	2.73 (1.78-4.18)	<0.001
Prephase Modification	7	0.18 (0.03-1.00)	4.3 (1.76-10.5)	0.001
Radiation	13	0.48 (0.26-0.89)	1.45 (0.64-3.29)	0.4
Intervention due to positive MRD	29	0.62 (0.46-0.84)	1.17 (0.64-2.15)	0.6

B.

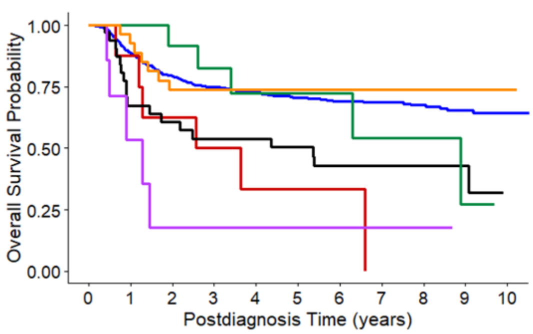

Number at risk

Protocol	603	412	352	315	272	226	185	147	112	71	35
Termination	4	7	5	4	2	1	1	0	0	0	0
Order Modification	20	20	18	16	16	14	11	6	5	4	0
Prephase Modification	7	3	1	1	1	1	1	1	1	0	0
Radiation	13	13	11	8	7	6	5	3	2	1	0
Intervention due to positive MRD	27	26	19	18	16	15	12	4	3	1	1

Type	N	5-yr OS (CI95)	HR (95CI)	P
Protocol	509	0.70 (0.66-0.75)	1.00	
Termination	10	0.33 (0.11-0.95)	3.20 (1.41-7.24)	0.005
Order Modification	35	0.50 (0.35-0.72)	2.31 (1.42-3.77)	<0.001
Prephase Modification	7	0.18 (0.03-1.00)	5.12 (2.09-12.54)	<0.001
Radiation	13	0.72 (0.50-1.00)	1.40 (0.57-3.42)	0.5
Intervention due to positive MRD	29	0.74 (0.59-0.92)	0.87 (0.41-1.86)	0.7

Supplementary Figure 5: Mantel Byar analyses; Simon Makuch plots of Disease-free survival (A) and Overall Survival (B) of patients in the trial ALL-REZ BFM 2002 in relation to the type of deviation; no deviation, preterm termination of the intensive chemotherapy, modification of the order of treatment courses, change of the cytoreductive prephase, modification of radiation plan, intensification due to MRD. pDFS, probability of disease-free survival; pOS, probability of overall survival; 95CI, 95% Confidence Interval; HR, hazard ratio

Reference:

1. Henze G, Charite University Berlin, Germany. ALL-REZ BFM 2002: Multi-Center Study for Children With Relapsed Acute Lymphoblastic Leukemia: ClinicalTrials.gov. Bethesda (MD): National Library of Medicine (US). 2000- [cited 2009 Jul 18]. NLM identifier: NCT00114348; 2005 [Available from: http://clinicaltrials.gov/ct2/show/NCT00114348
